# Central role of microglia in sepsis-associated encephalopathy: From mechanism to therapy

**DOI:** 10.3389/fimmu.2022.929316

**Published:** 2022-07-26

**Authors:** Xiaoqian Yan, Kaiying Yang, Qi Xiao, Rongyao Hou, Xudong Pan, Xiaoyan Zhu

**Affiliations:** ^1^ Department of Critical Care Medicine, The Affiliated Hospital of Qingdao University, Qingdao, China; ^2^ Department of Neurology, The Affiliated Hospital of Qingdao University, Qingdao, China; ^3^ Department of Neurology, The Affiliated Hiser Hospital of Qingdao University, Qingdao, China

**Keywords:** sepsis-associated encephalopathy (SAE), sepsis, microglia, cognitive impairment, inflammatory factors

## Abstract

Sepsis-associated encephalopathy (SAE) is a cognitive impairment associated with sepsis that occurs in the absence of direct infection in the central nervous system or structural brain damage. Microglia are thought to be macrophages of the central nervous system, devouring bits of neuronal cells and dead cells in the brain. They are activated in various ways, and microglia-mediated neuroinflammation is characteristic of central nervous system diseases, including SAE. Here, we systematically described the pathogenesis of SAE and demonstrated that microglia are closely related to the occurrence and development of SAE. Furthermore, we comprehensively discussed the function and phenotype of microglia and summarized their activation mechanism and role in SAE pathogenesis. Finally, this review summarizes recent studies on treating cognitive impairment in SAE by blocking microglial activation and toxic factors produced after activation. We suggest that targeting microglial activation may be a putative treatment for SAE.

## Introduction

According to expert consensus guidelines in 2016, sepsis is defined as a life-threatening organ dysfunction caused by the dysfunctional response of the body to a pathogenic infection (1). Brain dysfunction caused by sepsis is not associated with direct brain infection (2, 3) and occurs in approximately 70% of patients with sepsis (4, 5). Sepsis-associated encephalopathy (SAE) is a cognitive impairment associated with sepsis resulting in diffuse brain dysfunction without direct central nervous system infection or structural brain damage. Sepsis - associated encephalopathy may be the first symptom of sepsis (6), which is likely to have occurred before patients with sepsis were admitted to general wards and intensive care units (ICU) (6, 7). In an established murine model of sepsis, acute encephalopathy followed by long-term cognitive impairment, could be observed in the surviving mice (8). This long-term cognitive impairment was observed in more than half of the survivors, and their quality of life was significantly decreased (9).

Although attention has been brought to the harm caused by SAE, its pathogenesis remains unclear. There is a lack of clear diagnostic criteria and effective treatment measures. The SAE diagnostic criteria are based on the detection of electroencephalogram (EEG) abnormalities (10), cognitive impairment, and neuroimaging evaluation (6, 7). SAE onset is characterized by changes in the mental state that may range from delirium to coma. A wide range of neurological changes can be observed, such as impaired cognitive function and consciousness, inattention, personality changes, and the onset of depressive mood. Some patients have occasional tremors, stiffness, and EEG deviations (10, 11). Abnormal EEG waveform is related to the presence and severity of encephalopathy (12). Neuroinflammatory processes involve damage to the blood-brain barrier, pathways of inflammatory mediators, and activation of microglias, which amplify this process by releasing more inflammatory factors (13). Sedation is not a viable treatment owing to the complex pathophysiology of SAE (4, 14).

Therefore, the pathogenesis and pathophysiology of SAE must be comprehensively investigated to develop effective treatment measures to reduce the incidence of SAE and improve the quality of life of survivors. In this review, we describe the pathogenesis of SAE and demonstrate that microglia are closely related to the pathogenesis of SAE. Simultaneously, we systematically investigated the critical role of microglia in SAE, focusing on the phenotype, state, and function of microglia. Furthermore, we summarized the effects of inhibiting microglial activation or toxic factors after activation to alleviate cognitive impairment in SAE in recent years. Thus, it is reasonable to envisage SAE treatment by targeting microglia.

## Mechanism of sepsis-associated encephalopathy

The pathophysiological mechanisms of SAE, including various factors such as endothelial injury, inflammation, cerebral ischemia, blood-brain barrier (BBB) injury, and excitatory toxicity, remain unclear. Neuroinflammatory reactions, cerebral ischemic changes, and excitatory toxicity are common manifestations of severe sepsis (15). The comprehensive summary of several pathogenic mechanisms underlying SAE underscored the crucial role of microglia in these processes.

### Neuroinflammation

During sepsis, inflammatory factors and signals reach different regions of the brain through various means, such as body fluids and nerves (16). Neuroinflammation plays a vital role in the pathogenesis of SAE, as uncontrolled inflammatory responses are the main manifestations of sepsis. Additionally, neuroinflammation is the primary cause of brain dysfunction and apoptosis in brain cells (17).

When pro-inflammatory cytokines enter the brain, microglia are activated to release nitric oxide (NO), active nitrogen, and glutamate, further causing structural damage and inflammation of the cell membranes. Increased peroxynitrite production in the brain under the influence of NO and free radicals may affect brain cell function, further affecting glial cells, neurons, and the blood-brain barrier, leading to SAE-induced brain dysfunction (18–21). In vivo pro-inflammatory mediators promote the expression of brain endothelial cell adhesion molecules and active transport across the BBB through specific receptors, further promoting the entry of neurotoxic and inflammatory factors into the brain tissue (22–24). Pro-inflammatory cytokines that affect the brain include interleukin-1α (IL-1α), IL-1β, IL-6 (25, 26), and high mobility group box-1 protein (HMGB1). Tumor necrosis factor (TNF) passes through the BBB via tumor necrosis factor receptor 1 (TNF-R1) and TNF-R2 (27). As TNF is directly associated with BBB destruction, brain edema, neutrophil infiltration, astrocyte proliferation, and brain cell apoptosis, TNF may be a crucial mediator of SAE, and these manifestations do not occur in mice lacking the TNFR gene (28). In animal models, TNFR, IL-6, and IL-1 receptors antagonists (IL-1RA) are inversely associated with memory, suggesting that inflammatory factors are closely associated with cognitive impairment (29). In the later stages of sepsis, HMGB1 levels significantly increase in different brain regions (30). Antagonistic HMGB1 in the blood and brain regions can improve SAE by preventing damage to the brain cells and restoring neural cognitive function. This suggests that inflammatory cytokines are vital for the pathogenesis of SAE (30–32).

### Changes in cerebral ischemia and perfusion

In the pathological mechanism of sepsis, changes in blood flow and inflammatory responses may be critical steps in SAE development (33, 34). Impaired cerebral circulation during sepsis can lead to insufficient cerebral blood flow, which may be associated with electrophysiological and neurological changes (35, 36). Inadequate cerebral blood flow can lead to a cascade of cerebral ischemia which is controlled by three main processes: the reduction of oxygen and energy delivery (37), enhancement of stress signals (38), and activation of microglia (39). A decrease in energy supply can cause mitochondrial dysfunction, resulting in neuronal apoptosis and the release of pro-inflammatory cytokines (40). The increase of stress signals leads to the expression of adhesive molecules, which enhance the expression of matrix metalloproteinases (MMPs) signaling, which is related to an increase BBB permeability (41). Peripheral immune cells migrate to the brain and promote neuroinflammation (42, 43). Microglia can protect neurons to a certain extent as well as produce pro-inflammatory factors that cause neuronal apoptosis. Hemodynamic changes precede cognitive impairment and structural changes in the brain (14, 44, 45). Studies have shown that a continuous decrease in cerebral blood flow in patients with septic shock leads to impaired self-regulation and is associated with the onset of delirium (46). Neurovascular dysfunction is highly associated with decreased language and memory (47, 48). Maintaining the integrity of blood vessels in the brain is vital for cognitive function (34).

### Neurotransmitter dysfunction

During sepsis, the dopaminergic, β-adrenergic, GBAB receptors, and cholinergic nervous systems are impaired to a certain extent (15). An imbalance between the dopaminergic and cholinergic nerves is associated with cognitive impairment (49). This is related to the onset of SAE. Increased release of neurotransmitters, such as glutamate and acetylcholine, and reduced reuptake is one of the causes of neurotoxicity (50). Glutamate plays a role in neuronal apoptosis via excitatory toxicity (51). NO production may be related to neurotransmission disorders (52) or the excessive release of neurotoxic amino acids, such as ammonia, tyrosine, and tryptophan, into circulation by the liver and muscles during sepsis (53). Microglia express multiple neurotransmitter receptors, including glutamate, tyrosine, and acetylcholine. They also release glutamate, which induce neuronal apoptosis. Microglia communicate with each other and work together to regulate neuronal function.

### Disruption of the blood-brain barrier

The entry of aromatic amino acids into the brain through the damaged BBB leads to increased uptake of these amino acids by the brain (54), which further causes SAE, leading to an altered mental state (55). The BBB plays a vital role in stabilizing the brain milieu and maintaining adequate neural function by regulating the movement of ions and fluids between the blood and brain, thereby providing certain nutrients to the brain (56–58). The barrier also prevents external white blood cells from entering the central nervous system and playing an immunogenic role in the brain (59). Increased expression of complement activation, inflammatory cytokines (60, 61), and adhesion molecules further increase BBB destruction and helps white blood cells enter the brain, enhancing neuroinflammation.

During sepsis, inflammatory cytokines enter the central nervous system through various pathways, including receptor-mediated transcellular action, transcellular diffusion, and carrier proteins (62, 63).Proinflammatory cytokines such as IL-1β, IL-6, and lipopolysaccharide, reactive oxygen species, and NO act on BBB to alter brain function, resulting in the disruption of brain homeostasis and changes in BBB permeability (64–66). These inflammatory factors enter the brain tissue and activate microglia (49). Prior to the change in BBB permeability, microglia begin to migrate to the cerebrovascular site and respond to the surrounding inflammatory factors, which play a certain protective role in the BBB in the early stage (67). However, further inflammation leads to a more active microglial phenotype, increased phagocytosis of astrocyte terminal feet, and increased BBB permeability. Microglia can decrease paracellular connexins expression and further enhance BBB permeability.

**Figure 1:**
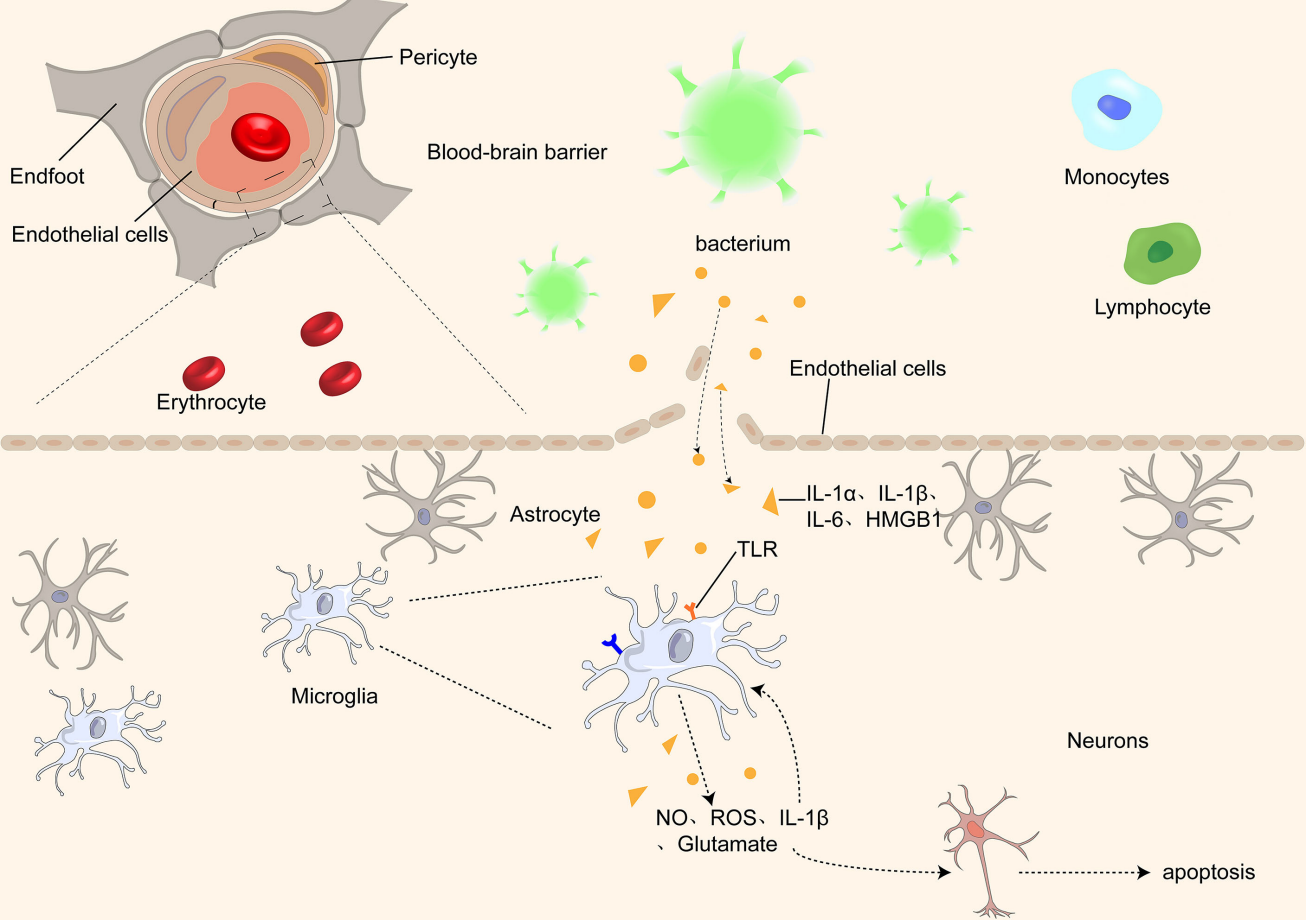
Proinflammatory cytokines, such as IL-1α, IL-1β, IL-1, and HMGB1, pass through the BBB through different receptors and activate microglia, which further damage the BBB by releasing inflammatory factors, causing an inflammatory cascade. At the same time, microglia release ROS and NO, causing damage and even apoptosis of neurons in the brain.

Overall, the pathogenesis of SAE is not caused by a single factor but by the joint action of multiple factors. (**Figure 1**) Microglial activation is essential in the pathogenesis of SAE. It is involved in almost every stage of SAE pathogenesis. Moreover, it interacts with various central nervous system components and plays a vital role in maintaining brain function and integrity. Furthermore, it is closely associated with cognitive dysfunction in central nervous system diseases; therefore, we will focus on the relationship between microglia and cognitive dysfunction in different nervous system diseases.

## Function, phenotype, and the role of microglia in SAE

Microglia play a vital role and are closely related to SAE pathogenesis. Therefore, we focused on the function, phenotype, and phenotypic transformation of microglia. Finally, the activation of microglia and their role in SAE development are highlighted in this section.

### Function of microglia

**Figure 2:**
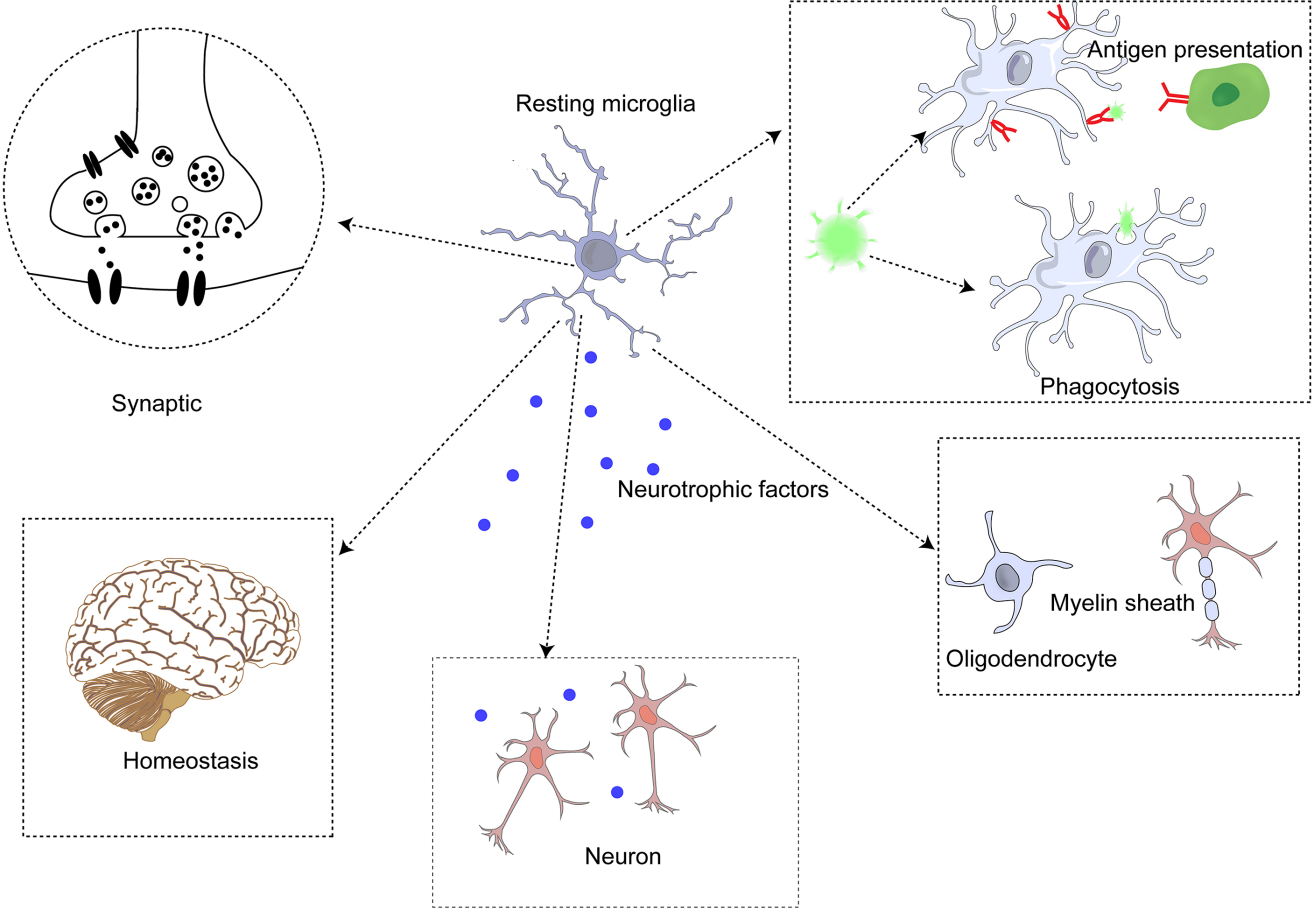
Microglia perform functions in the central nervous system.

Microglia are the primary immune cells in the brain parenchyma and differ from other macrophages in the brain (68). Studies of ApoE4 alleles showed that microglia play an important role in neurodegenerative diseases. E4-expressing microglia showed higher innate immune reactivity after LPS treatment, significantly reduced neuronal activity, and secreted more elevated levels of TNF when co-cultured with neurons (69). Microglia perform some critical functions in the brain (70) synaptic genesis, modification, and plasticity changes (71); (2) detection of local steady state (72); (3) immune function, including phagocytosis, antigen presentation, secretion of anti-inflammatory (such as IL-10, IL-4) and pro-inflammatory (such as IL-1β, IL-6) cytokines, and regulation of neuronal apoptosis (73); (4) regulation of myelin sheath (74); (5) neurotrophic support (75); and (6) communicate with astrocytes to regulate these functions (76). (**Figure 2**) Furthermore, microglia are involved in synapses and neurogenesis, as well as in the removal of unwanted neuronal and other cellular waste. Monitoring the changes in the brain microenvironment suggested that microglia alter both in shape and function through microglial activation (77).

Microglia exist in resting and active states (78). In dynamic homeostasis, microglia secrete neurotrophic factors and monitor the microenvironment with scavenger receptors (SR) to remove unnecessary cell debris and apoptotic neurons, thus further maintaining the homeostasis and connection of neuronal functions (72, 79). Microglia in dynamic equilibrium are rod-shaped with many forked processes (73). Although they are in equilibrium, they still monitor the state of their microenvironment and surrounding tissues to acutely alert them to abnormal signals. The morphology of microglia changes significantly after activation, the cell body is enlarged, and the process is shortened (68). The status of microglia can be assessed by their movement and morphology in different environments (77).

### Microglial phenotypes

**Figure 3:**
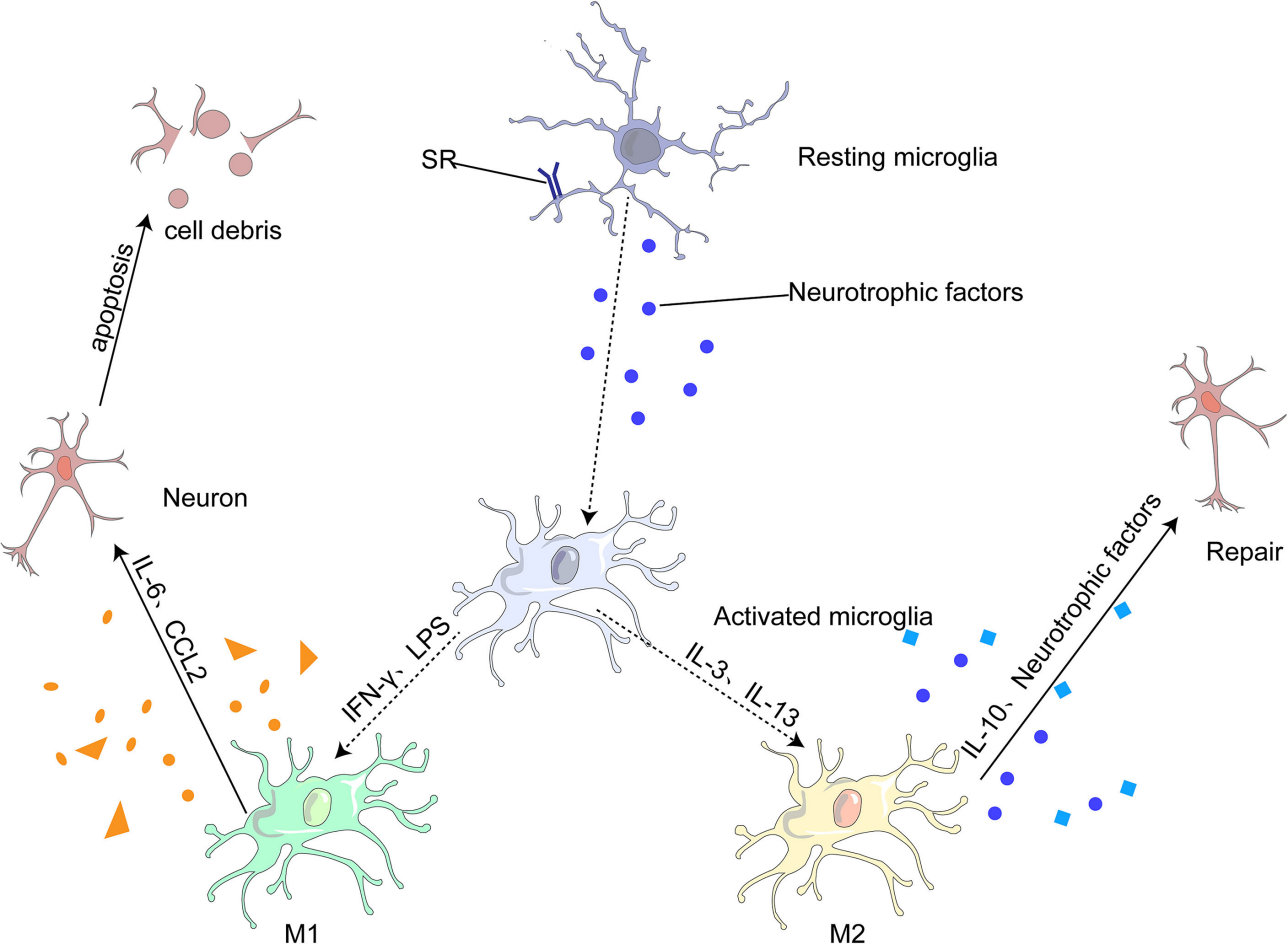
Most microglia can be divided into two opposite types: classical (M1) or alternative (M2). M1 microglia release inflammatory mediators, such as IL-6 and CCL2, which induce inflammation and neurotoxicity. M2 microglia release anti-inflammatory mediators, such as IL-10 and neurotrophic factor, which induce anti-inflammatory and neuroprotective effects.

Under different microenvironments, microglia undergo produce phenotypic changes, including the M1 pro-inflammatory, M2 anti-inflammatory and other phenotypes (75, 80). M1 microglia can lead to neuroinflammation and neuronal apoptosis, whereas M2 microglia can protect neurons and repair brain tissues. M1 is generally activated by interferon-γ (IFN-γ) and lipopolysaccharide (LPS) to produce IL-6, and CC-chemokine ligand 2 (CCL2), which ultimately leads to neuronal damage and even apoptosis (68, 81). M2 microglia are generally induced by anti-inflammatory cell mediators, including IL-13 and IL-3. It produces IL-10 and neurotrophic factors to repair brain tissue and neurons (68, 75). (**Figure 3**)

Microglia can change their phenotype from M1 to M2 in the following ways (82): (1) different signaling pathways, such as the Toll-like receptor signaling pathway (83), Janus kinase/signal transducer and activator of transcription (JAK/STAT) signaling pathway (84, 85), NF-κβ signaling pathway (86, 87), and mitogen-activated protein kinase signaling pathway (88); and (2) regulatory transcription factors, such as PPAR-γ (89), which exert anti-inflammatory effects by inhibiting NF-κβ, STAT, and other transcription factors. These transcription factors are closely associated with microglial polarization into type M1, a transcription pro-inflammatory factor. Inhibition of these factors increased the polarization of microglia from M1 to M2; (3) regulation of microglial surface receptors, such as TREM2 (90)and α7 nicotinic acetylcholine receptor (α7nAChR) (91); (3) regulation of different cytokines, such as IL-4 (92), IL-10, TGF-β (75), neurotrophic factor; (5; change in channels (93); (6) bioactive compounds and certain drugs (94–96). The transformation of microglia from the M1 to M2 phenotype is not caused by a single process described in this section but by a combination of several different mechanisms.

Microglial activation damages nearby healthy brain tissue, while the affected nerve tissue may secrete substances that, in turn, promote microglial activation. The activation of microglia by chronic inflammation in the human body increases the occurrence of synapses, resulting in enhanced phagocytosis and neuronal apoptosis (97). Microglia, supported by neurotrophic factors, contribute to the formation of synapses associated with learning and memory, which are implicated in cognitive function (98).

### Microglia in CNS-related cognitive impairment

Microglial dysfunction, which affects the structure and function of the brain, is associated with almost all brain diseases, including neurodegenerative diseases (such as Alzheimer’s disease [AD], stroke, and Parkinson’s disease), as well as inflammatory brain diseases (99, 100), that can cause long-term cognitive impairment (97).

Microglia are carriers of amyloid precursor protein and promote Aβ production in rat brain tissue, which is closely related to AD (101). Microglia can bind to Aβ by expressing the corresponding receptors CD36, TLR2, and TLR4, induce the release of IL-1β, and trigger neuroinflammation (102). In AD mouse models, TLR and IL-1β deficiency can reduce Aβ deposition and prevent cognitive impairment (102). Datta et al. believed that in neurodegeneration after stroke, an increase in misfolded proteins and microglial activation can be found in the thalamus, leading to neuronal loss and further deterioration of cognitive function (103). Activation of microglia and increased pro-inflammatory factors are also important mechanisms in Parkinson’s disease (PD) (104, 105). Microglia were significantly active in the substantia nigra pars compacta in the PD murine model, and the secretion of inflammatory cytokines in this region was also significantly enhanced. These inflammatory cytokines can cause neuronal damage and even apoptosis (106) and lead to the degeneration of dopaminergic neurons (107), resulting in cognitive dysfunction. Simultaneously, the function of microglia declines with the increase age (68). The immune receptor expression is increased along with the release of more neurotoxic substances, which goes hand to hand with neurodegenerative diseases (108–110). These studies suggest that the activation of microglia is one of the crucial links in the pathogenesis of AD, stroke, and PD (105, 106, 111). Notably, we also observed that microglial activation was strongly associated with cognitive impairment in patients.

Microglia play a vital role in the pathogenesis of neurodegenerative diseases and in infectious encephalopathy. In studies of viral encephalitis diseases (77) (such as Japanese encephalitis, West Nile and Zika viruses), INF-γ induced microglial activation, then produced different inflammatory signals such as IL-1, and IL-6, which can directly cause neurotoxic lesions. It can also lead to cognitive impairment and neuronal over-firing (112–114).

Treatment of intracranial malignant tumors targets cancer cells and leads to the activation of microglia, which changes from a neurotrophic to neurotoxic state (115). In a glioma mouse model, cognitive impairment is associated with microglial activation induced by repeated cranial irradiation rather than the tumor itself (116). These findings suggest that microglial activation can lead to cognitive dysfunction in central nervous system diseases.

### Microglia in SAE

After activation, microglia can cause neuron injury or even apoptosis by releasing inflammatory mediators, reactive oxygen species, neurotransmitters and other substances, which play an important role in the pathogenesis of SAE (Fig 1). Chemokines and inflammatory cytokines secreted by microglia can help the brain defend against inflammatory responses, regulate the migration of white blood cells, and facilitate the repair of neurons in brain tissue (117). However, long-term microglia activation has a minimal protective effect on neurons and further worsen the inflammatory response in the brain. The most common SAE models are divided into two types: intraperitoneal injection of LPS and caecal ligation perforation (CLP) (118, 119). An increase in the number of ED-1-positive microglia was observed 24 h after establishing an SAE mouse model by LPS injection. They are distributed around the cerebrovascular system and around the parenchyma. Most microglia are reportedly distributed around cerebral vessels 4h after LPS injection. The extent of microglial activation was time-dependent, and the highest microglia numbers were observed at 8h in all brain regions (20, 120). The production of TNF was induced by LPS stimulation in vitro (121). LPS strongly stimulates microglia activation, and disordered activation of microglia during SAE may lead to further deterioration of already damaged brain tissue (119, 122). The establishment of CLP model showed a significant increase in the size and number of microglial processes by immunofluorescence (121). Simultaneously, the SAE model established by CLP can also cause microglial overactivation and neuronal pyroptosis, aggravating brain tissue destruction and cognitive dysfunction (118).

Peripheral circulating inflammatory factors induce immune-related responses in the central nervous system through various pathways, including inflammatory mediators (42, 49), adjacent cells (123, 124), and neurotransmitters (49, 125), which may be strongly linked to the role of microglia in sepsis. In particular, microglia are activated through the pathways discussed earlier, resulting in neuronal damage and even apoptosis, further leading to SAE.

As described above, microglial activation is strongly associated with the occurrence of SAE. Microglia can recognize various damage signals, including microorganisms, complements, and cytokines. They are thought to injury to the central nervous system (126). Microglia are activated by bacteria and other substances through Toll-like receptors (TLR-2, TLR-4, and TLR-9) and nucleotide-binding oligonucleotide 2 (NOD2) (42). Simultaneously, inflammatory cytokines such as IL-1β and IL-6 activate microglia through the damaged BBB, leading to brain cell destruction and even apoptosis during sepsis (49). IL-17A/IL-17R signaling pathway forms a vicious inflammation cycle and amplifies the role of inflammation in the brain by promoting the secretion of inflammatory factors by microglia and intensifying IL-17A secretion by immune cells (42, 125). Adjacent cells, such as astrocytes, endothelial cells, and Th1/Th17 cells, have regulatory effects on microglia. Astrocytes attach to vascular endothelial cells or via meningeal cells in peripheral blood vessels and express multiple cytokine receptors that enable astrocytes to respond to inflammation (123). Microglia are activated by bacteria and other substances through cytokines secreted by astrocytes, such as granulocyte colony-stimulating factor (G-CSF) and CCL11. G-CSF is a microglial growth factor, and CCL11 can promote microglial migration to inflammatory sites, causing microglia to produce reactive oxygen species, resulting in the destruction and even apoptosis of brain cells (123). Astrocytes activate microglia and induce apoptosis of brain cells as well as produce anti-inflammatory substances that inhibit inflammation in the central nervous system (127). CX3CR1 is a chemokine and transmembrane protein that promotes leukocyte migration in monocytes, dendritic cells, and microglia (128). CX3CR1 is a receptor for CX3CL1; the interaction between CX3CL1 in neurons and CX3CR1 in microglia mediates the functional phenotype of microglia and its overactivation under inflammatory conditions. Increased CX3CL1 expression on endothelial cells activates endothelial cells and promote increased leukocyte adhesion, microthrombus formation, coagulation disorders, and metastasis of microglia to inflammatory sites (124, 125). Th1/Th17 cells produce large amounts of IL-17A in the brain, inducing microglial activation and prolonging inflammatory processes (43). Microglia express receptors for various neurotransmitters, including glutamate and acetylcholine, and communicate with each other to maintain normal neuronal function (129). During sepsis, an imbalance in the expression of different types of neurotransmitters, such as glutamate and acetylcholine, affects the function of microglia and neurological function. Activated microglia can produce several inflammatory factors around the cerebrovascular or cerebral solid, leading to an enhanced brain immune response, further causing neuronal damage, loss of function, and even apoptosis (130, 131).

Previous studies have shown that in case-control studies of patients who died of sepsis, CD68 expression was significantly elevated in the cortex of the experimental group compared to that in the control group, and deformed microglial cells were also observed (132, 133). Activated microglia can induce neurological dysfunction and memory loss in patients with sepsis by releasing pro-inflammatory cytokines and including the expression of related enzymes (134). Microglia regulate neuronal function through neurotransmitters levels. In a postmortem case-control study of patients with delirium, the expression of microglial markers CD68 and HLA-DR were significantly increased compared to that in the controls, suggesting that microglial activation may be associated with delirium (135). Microglial activation has also been detected during sepsis (136–138). During sepsis, intraventricular injections of minocycline inhibit microglia and reduce acute brain injury, inflammation, and long-term cognitive impairment in survivors (139).

Therefore, when activated microglia sense surrounding injury signals, the cells may be more prone to release several inflammatory cytokines, resulting in cognitive dysfunction and exacerbation of SAE. Further exploration of the role of microglial activation in SAE cognitive dysfunction can deepen our understanding of the pathogenesis of SAE and may provide evidence for the treatment of SAE.

## Targeting microglia to treat SAE cognitive impairment

The activation of microglia, as a central link in the development of cognitive deficits in sepsis-associated encephalopathy, could represent an effective therapeutic target (140). Several studies have shown that blocking microglia activation or alleviating a series of neurotoxic reactions after microglia activation can improve neurological symptoms and long-term cognitive dysfunction to a certain extent (42, 139, 141). Based on this concept, we have summarized the potential value of targeting microglia in diagnosing and treating of SAE cognitive impairment.

### Prevents microglial activation

Inflammatory mediators, neurotransmitters, and intercellular interactions with surrounding cells accelerate microglial activation (125). Once activated, inflammatory factors, reactive oxygen species, NO, prostaglandins, and neurotoxic glutamate continue to act on the neighboring neurons, causing neuronal damage and, ultimately, cognitive impairment (15, 142). Therefore, early identification and prevention of microglial activation are crucial.

Aseptic neuroinflammation caused by circulating inflammatory mediators in the brain has long been accepted as the pathogenesis of SAE. However, a study on the intestinal flora in septic encephalopathy found that in the absence of evident sepsis, the bacteria temporarily translocate to the brain and cause microglial activation and neuroinflammatory responses (121). Activation of the host immune mechanism may cause persistent cognitive dysfunction. Therefore, early attention to specific microbiota may later improve cognitive dysfunction (121, 143). A recent study by Zhang et al. (143) demonstrated for the first time the exact relationship between gut microbiota and its metabolite butyric acid and SAE. SAE mouse models of different severity were constructed by CLP and fecal microbiota transplantation(FMT) was performed on sterile mice, confirming the significant role of the gut-brain axis in SAE. In particular, butyrate has been found to reduce oxidative stress response and nerve damage through the GPR109A receptor on microglia and the Nrf2/HO-1 signaling pathway. Furthermore, recent sequencing analysis of microglia and brain endothelial cells revealed endotheliitis as the earliest microglial activation event. Microglia are activated by cerebral endothelial cells (CECs)-derived inflammatory mediators. Therefore, early recognition and blocking of CECs activation can also reduce microglial activation and subsequent reactions (144).

Sirtuins have been widely studied as long-lived proteins (145, 146). Sirt3 is a mitochondrial enzyme that plays a vital role in the metabolic cycle and participates in the regulation of apoptosis. High levels of Sirt3 were detected in LPS-induced mouse microglia. In contrast, the Sirt3 levels decreased after treatment with Single-wall carbon nano horns (SWNHs), which delayed the mitosis of microglia and promoted their apoptosis. Therefore, SWNHs may be a therapeutic approach to inhibit microglial activation by blocking Sirt3 (147). Another study used resveratrol (a SIRT1 activator) to induce SIRT1 overexpression, which plays an important role in inflammatory regulation, inhibiting microglial activation and proliferation, as well as inflammatory processes in SAE mice (148). Shi et al. (149) observed that SIRT1 regulates oxidative stress in hypoxic and glucose-deficient hippocampal neurons and has a protective effect on nerve cells after oxygen and glucose deprivation (OGD). These studies have revealed the crucial role of SIRT1 in microglial activation and neuronal protection.

TLR4, an immune recognition receptor, is highly expressed in LPS-induced microglia and is closely associated with neuroinflammation through a cascade of downstream pathways after activation (150, 151). The exposure TLR4 to G+ bacterial LPS activates a series of downstream proteins, one of which ultimately activates NF-κβ, initiating transcription and producing a pro-inflammatory effect. In septic mice treated with sodium butyrate (NaB), activation of hippocampal microglia and secretion of inflammatory factors were reduced, and improvements in neuroinflammation and anxiety were observed. The mechanism underlying these benign results is due to NaB antagonization of TLR4 activation, consequently inhibiting subsequent nuclear transcription (152). Protein kinase C-interacting protein (PICK1) is the most abundant protein in the brain and plays a unique role in the progression of many neurological diseases. In addition, PICK1 is involved in several inflammatory pathological processes (153, 154). Wang et al. observed overactivated microglia, TLR4 pathway, and PICK1/TLR complex in an SAE mouse model with a PICK1 knockout. However, PICK1 levels were not significantly altered in LPS-induced sepsis mice. They demonstrated for the first time that PICK1 plays a protective role in SAE by forming a complex with TLR4 (144). Another study used electroacupuncture to improve neural function, possibly by increasing the PICK1/TLR4 complex in microglia to provide protection (155).

IL-17A is reportedly involved in this acute cycle of microglial activation. Adjacent cells, such as CD4+T cells and Th17 cells, secrete IL-17A, and act on the surface receptors of microglia, thereby activating them. Activated microglia secrete several inflammatory factors, including IL-17A, which undoubtedly aggravate microglial activation (156, 157). By injecting recombinant IL-17A, anti-IL-17A antibody, and anti-IL-17R antibody into CLP mouse models, Ye et al. (42) revealed the potential role of IL-17A/IL-17R blockade in preventing SAE.

Blocking the activation of SAE microglia by blocking inflammatory factors, signaling pathways, and other pathways is of great significance for the early prevention of SAE cognitive impairment. Further studies are warranted in this regard.

### Reduce neuronal injury after activation of microglia

Once microglia are activated, their neurotoxic effects accelerate the progression of SAE and are strongly associated with long-term cognitive impairment (140). Survivors of sepsis-associated encephalopathy are at a higher risk of developing dementia, and long-term cognitive impairment is considered a transitional state before the onset of dementia (158). Therefore, improving cognitive impairment is essential for the outcome of patients with SAE, and this process can be achieved by reducing the neurotoxicity of activated microglia. Based on this pathogenesis, we believe that inflammation, oxidative stress, apoptosis, and immune response can reduce microglial neurotoxicity and improve cognitive impairment. Memory disorders are mainly dominated by neuroinflammation in the hippocampus, and IL-1 β levels are negatively correlated with the severity of memory disorders (159). Activated microglia can release many cytokines including IL-1β. Therefore, regulating the inflammatory response after activation of microglia may play a role in improving a range of cognitive disorders such as memory impairment.

Water maze and fear conditioning tests were performed on SAE mice constructed by cecal ligation and perforation (CLP). Impaired learning and memory functions were observed, whereas C-X-C chemokine receptor type (CXCR) 5 expression was upregulated. When CXCR5 was knocked out, cognitive deficits and M1 polarization were reversed, and similar results were observed in primary microglia in vitro. Downregulation of CXCR5 reduces the pro-inflammatory microenvironment in the hippocampus, which may be a potential therapeutic target (160). Previous studies have shown that inflammasome activation is essential in SAE, with NOD, LRR and pyrin domain-containing protein 3 (NLRP3) inflammasome being the most representative. Resveratrol inhibits the NLRP3/IL-1β axis of microglia, reduces hippocampal inflammation, and improves spatial memory in SAE mice (161). In another study, treatment with ethyl pyruvate significantly reduced cognitive impairment in CLP mice by inhibiting NLRP3 and inducing IL-1β cleavage (162). In conclusion, inhibition of NLRP3 can potentially improve cognitive impairment in SAE.

Stanniocalcin-1 (STC-1), a neuroprotective protein, plays an anti-inflammatory and antioxidant role by inducing the uncoupling proteins (UCPs). Injection of recombinant human STC-1 (rhSTC1) inhibited microglia production of pro-inflammatory factors and improves cognitive impairment (163). Moreover, the positive effects of NOS2 gene deletion and propofol inhibition of NMDA receptors on cognitive impairment in sepsis-associated encephalopathy are achieved by inhibiting microglial inflammation (134, 164).

Activating the nuclear factor erythroid 2-related factor 2 (Nrf2) signaling pathway is beneficial to SAE. In addition to mediating the inactivation of NLRP3 and playing an anti-inflammatory role, Nrf2 acts as an endogenous antioxidant and plays a neuroprotective role (165). H2 protects neurons from activated microglia by upregulating the Nrf2 pathway and antagonizing oxidative stress (166). In an experiment on the effect of ginsenoside on SAE, it was found that ginsenoside inhibited oxidative stress and apoptosis, and the mechanism was related to the upregulation of Nrf2 and heme oxygenase-1(HO-1) (167).

Collectively, reducing the toxic effects of microglial activation is of great significance in treating cognitive impairment in sepsis-associated encephalopathy. It provides a new idea to design a treatment for SAE cognitive impairment by targeting various pathways in microglia.

## Conclusion

SAE is associated with increased mortality in patients with sepsis and reduced quality of life in survivors; therefore, further research is required to treat cognitive impairment in SAE. In this review, we have provided a comprehensive overview of the different functions and phenotypes of microglia, and their role in SAE pathogenesis. Notably, we summarized recent advances in treating of cognitive impairment in SAE based on microglial activation and the associated toxic effects of microglia activation. We are confident that further research on microglia will provide novel insights into the treatment of SAE.

## Author contributions

XY, KY and QX were involved in reading and editing the manuscript and all authors commented on previous versions of the manuscript. All authors read and approved the final manuscript.

## Conflict of interest

The authors declare that the research was conducted in the absence of any commercial or financial relationships that could be construed as a potential conflict of interest.

## Publisher’s note

All claims expressed in this article are solely those of the authors and do not necessarily represent those of their affiliated organizations, or those of the publisher, the editors and the reviewers. Any product that may be evaluated in this article, or claim that may be made by its manufacturer, is not guaranteed or endorsed by the publisher.
